# Magnesium Deficiency Induces Lipid Accumulation in Vascular Endothelial Cells via Oxidative Stress—The Potential Contribution of EDF-1 and PPARγ

**DOI:** 10.3390/ijms22031050

**Published:** 2021-01-21

**Authors:** Laura Locatelli, Giorgia Fedele, Sara Castiglioni, Jeanette A. Maier

**Affiliations:** 1 Department Biomedical and Clinical Sciences L. Sacco, Università di Milano, Via GB Grassi 74, 20157 Milano, Italy; laura.locatelli@unimi.it (L.L.); giorgia.fedele@unimi.it (G.F.); sara.castiglioni@unimi.it (S.C.); 2 Interdisciplinary Centre for Nanostructured Materials and Interfaces (CIMaINa), Università di Milano, 20133 Milano, Italy

**Keywords:** magnesium, endothelial cells, EDF-1, PPARγ, lipids

## Abstract

Background: Magnesium deficiency contributes to atherogenesis partly by promoting the dysfunction of endothelial cells, which are critical in vascular homeostasis and diseases. Since EDF-1 and PPARγ regulate crucial endothelial activities, we investigated the modulation of these proteins involved in lipogenesis as well the deposition of lipids in human endothelial cells cultured in different concentrations of magnesium. Methods: Human endothelial cells from the umbilical vein were cultured in medium containing from 0.1 to 5 mM magnesium for 24 h. The levels of EDF-1 and PPARγ were visualized by Western blot. Reactive oxygen species (ROS) were measured by DCFDA. Lipids were detected after O Red Oil staining. Results: Magnesium deficiency leads to the accumulation of ROS which upregulate EDF-1. Further, PPARγ is increased after culture in low magnesium, but independently from ROS. Moreover, lipids accumulate in magnesium-deficient cells. Conclusions: Our results suggest that magnesium deficiency leads to the deposition of lipids by inducing EDF-1 and PPARγ. The increase in intracellular lipids might be interpreted as an adaptive response of endothelial cells to magnesium deficiency.

## 1. Introduction

Vascular endothelial cells (EC), which constitute the thin layer lining the inner surface of blood vessels, are crucial to maintain the integrity of the vascular wall and, therefore, the homeostasis of the entire organism. Besides affecting the traffic of molecules and solutes between the blood and neighboring tissues, EC regulate blood fluidity, vascular tone, leukocyte trafficking and immune response. In case these functions are dysregulated, EC acquire a pro-oxidant and pro-inflammatory phenotype, thus promoting cardiovascular diseases [1].

Mechanical forces [2] as well as a multitude of different soluble molecules induce endothelial dysfunction [1]. Alterations of magnesium (Mg) homeostasis contribute to cardiovascular diseases [3] and, in particular, to atherogenesis. Indeed, low Mg induces the acquisition of a pro-atherogenic phenotype in EC *in vivo* and *in vitro*. Mg deficiency facilitates the uptake of low-density lipoproteins by EC, impairs their antioxidant responses, promotes senescence and models gene expression [4,5,6]. In cultured human macrovascular endothelial cells, Mg deficiency induces the acquisition of a pro-inflammatory phenotype [7,8] and it is well known that inflammation is a hallmark of atherogenesis [9].

Endothelial differentiation-related factor-1 (EDF-1) is a low-molecular weight intracellular protein, which is ubiquitously expressed and highly conserved during evolution [10]. EDF-1 exerts its function both in the cytosol and in the nucleus. In the cytosol, it binds calmodulin [11,12,13], a Ca(2+)-binding protein which modulates several calcium-regulated enzymes, among which is the endothelial NO synthase (eNOS) [14]. In addition, EDF-1 is required for various events associated with ribosome-mediated quality control pathways [15].

In the nucleus, EDF-1 interacts with several transcription factors, among which is the TATA Binding Protein (TBP), and acts as a transcriptional coactivator for non-steroid nuclear receptors involved in lipid metabolism [16,17], among which is the peroxisome proliferator-activated receptor gamma (PPARγ), a nuclear receptor regulating transcription of several genes implicated mainly in fatty acid and energy metabolism [18]. In the endothelium, PPARγ modulates cytokines production, proliferation, migration, energy metabolism, apoptosis and angiogenesis [19,20,21]. Of notice, PPARγ inhibits the expression of pro-inflammatory molecules and the activities of other transcription factors, such as activator protein-1 (AP-1) and nuclear factor (NF)-kB [22]. Accordingly, the activation of PPARγ inhibits the expression of the adhesion molecules VCAM-1 and ICAM-1 in activated endothelial cells *in vitro* and markedly reduces monocyte/macrophage homing to atherosclerotic plaques *in vivo* [23]. In high-fat diet-fed apolipoprotein E-deficient mice, PPARγ protects against IL-1β-mediated endothelial dysfunction through a reduction in oxidative stress responses [24].

Since (i) Mg deficiency promotes endothelial dysfunction [5,6] and (ii) EDF-1 plays a role in controlling endothelial performance [25], we investigated Mg-dependent modulation of EDF-1 in primary human EC.

## 2. Results

### 2.1. Low Magnesium Upregulates EDF-1

**Figure 1:**
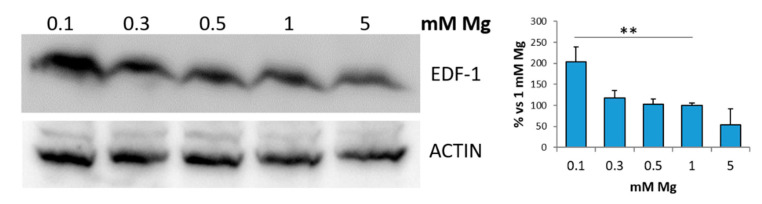
Low magnesium induces EDF-1. HUVEC were cultured in medium containing 0.1, 0.3, 0.5, 1 or 5 mM of extracellular Mg for 24 h. Cell extracts were processed for Western blot using antibodies against EDF-1. Actin was used as a control of loading. A representative blot is shown. Densitometry (right panel) was performed on three different blots using Image J. ** *p* ≤ 0.01.

Initially, we examined the amounts of EDF-1 in human endothelial cells from the umbilical vein (HUVEC) cultured for 24 h in medium containing different concentrations of extracellular Mg, i.e., low (0.1, 0.3 and 0.5 mM), physiological (1 mM) and high (5 mM) Mg. Figure 1 shows a significant increase in EDF-1 in cells maintained in 0.1 mM Mg compared to the control in 1 mM Mg or to cells in 5 mM Mg.

### 2.2. Low Magnesium Induces Oxidative Stress in HUVEC

**Figure 2:**
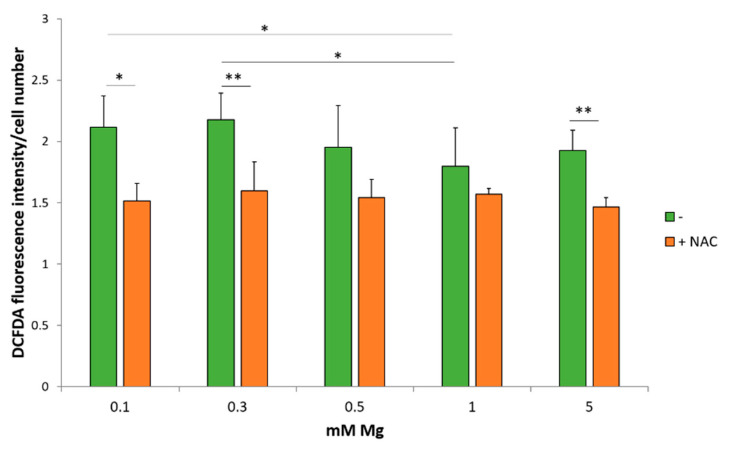
Low magnesium induces ROS accumulation. ROS production was evaluated using DCFDA in HUVEC maintained for 24 h in media containing different concentrations of Mg in the presence (orange columns) or in the absence (green columns) of NAC (5 mM). Fluorescence at 529 nm was measured and then normalized to the cell number. The experiment was performed three times in triplicates. * *p* ≤ 0.05 and ** *p* ≤ 0.01.

In *in vitro* models, Mg deficiency has been linked to oxidative stress [26,27]. Of note, EDF-1 plays a protective role in oxidative stress in Drosophila [28]. Consequently, we measured ROS production in HUVEC cultured for 24 h in medium containing different concentrations of extracellular Mg. The 2’-7’-dichlorofluorescein diacetate (DCFDA) solution reveals that HUVEC produce more ROS when cultured in medium containing 0.1 and 0.3 mM Mg compared to the physiological or high concentrations of the cation (Figure 2, green columns). This effect is prevented by pretreating the cells with the glutathione precursor N-Acetil-Cysteine (NAC) (Figure 2, orange columns) [29]. NAC also reduces ROS in HUVEC cultured in 5 mM Mg. 

### 2.3. EDF-1 Is Induced by Oxidative Stress

**Figure 3:**
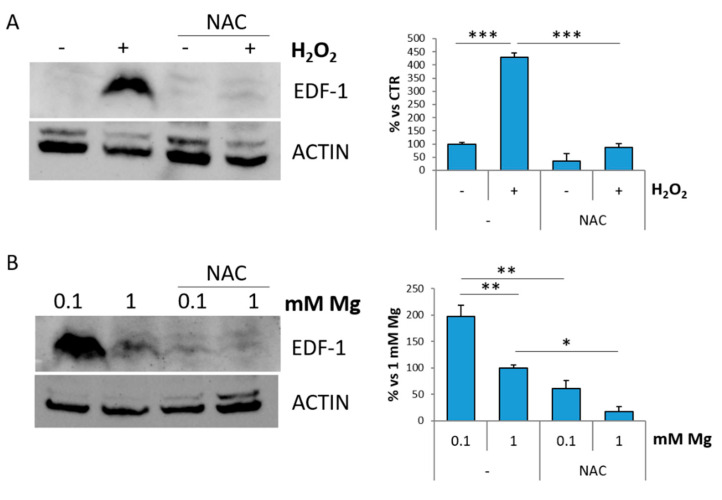
Oxidative stress induces EDF-1. (**A**) HUVEC were pretreated or not with NAC (5 mM) for 1 h, exposed or not to H_2_O_2_ (200 μM) for 30 min and extracted 24 h later. (**B**) HUVEC were cultured in 0.1 or 1 mM of Mg in the presence or in the absence of NAC (5 mM) for 24 h. Western blot was performed using antibodies against EDF-1. Actin was used as a control of loading. A representative blot is shown. Densitometry (right panel) was performed on three different blots using Image J. * *p* ≤ 0.05, ** *p* ≤ 0.01 and *** *p* ≤ 0.001.

To assess whether oxidative stress modulates EDF-1, we treated HUVEC for 30 min with H_2_O_2_, a strong inducer of oxidative stress, and then maintained the cells in culture for 24 h. The MTT assay reveals that the cells are viable (data not shown). Western blot shows a significant increase in EDF-1 levels prevented by NAC (Figure 3A). Then, HUVEC were cultured in 0.1 or 1 mM Mg with or without NAC for 24 h. As shown in Figure 3B, NAC prevents low Mg-induced increase of EDF-1, confirming the direct role of oxidative stress in the induction of EDF-1.

### 2.4. Low Magnesium Induces PPARγ

**Figure 4:**
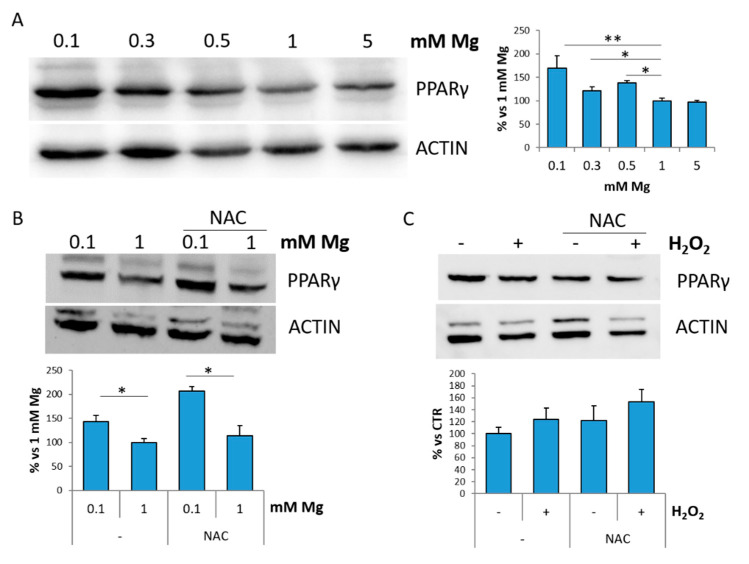
Low magnesium induces PPARγ. (**A**) HUVEC were cultured in low (0.1, 0.3, 0.5 mM), physiological (1 mM) and high (5 mM) extracellular Mg for 24 h. (**B**) HUVEC were cultured in 0.1 or 1 mM in the presence or in the absence of NAC (5 mM) for 24 h. (**C**) HUVEC were treated with H_2_O_2_ (200 μM) for 30 min in the presence or in the absence of NAC (5 mM) and then maintained in culture for 24 h. Cell extracts were processed for Western blot using antibodies against PPARγ. Actin was used as a control of loading. A representative blot is shown. Densitometry (right panel) was performed on three different blots by Image J. * *p* ≤ 0.05 and ** *p* ≤ 0.01.

EDF-1 is a transcriptional activator for PPARγ [16,17,18]. We analyzed the total amounts of PPARγ in HUVEC cultured in the presence of various concentrations of Mg for 24 h and found that PPARγ levels are increased in 0.1, 0.3 and 0.5 mM Mg, while they are comparable in HUVEC cultured in physiological or high Mg (Figure 4A). We focused on HUVEC in 0.1 mM Mg to test if ROS are implicated in increasing PPARγ in the low-magnesium condition. To this purpose, we incubated the cells cultured in 0.1 or 1 mM Mg with NAC. Figure 4B shows that NAC does not prevent PPARγ accumulation in Mg-deficient HUVEC. We then exposed HUVEC to H_2_O_2_ for 30 min and maintained them in culture medium for 24 h. No modulation of the levels of PPARγ in response to H_2_O_2_ with or without NAC is observed by Western blot (Figure 4C). These results suggest that ROS are not involved in modulating PPARγ in cells cultured in low extracellular Mg.

### 2.5. Low Magnesium Induces Lipid Accumulation in HUVEC

**Figure 5:**
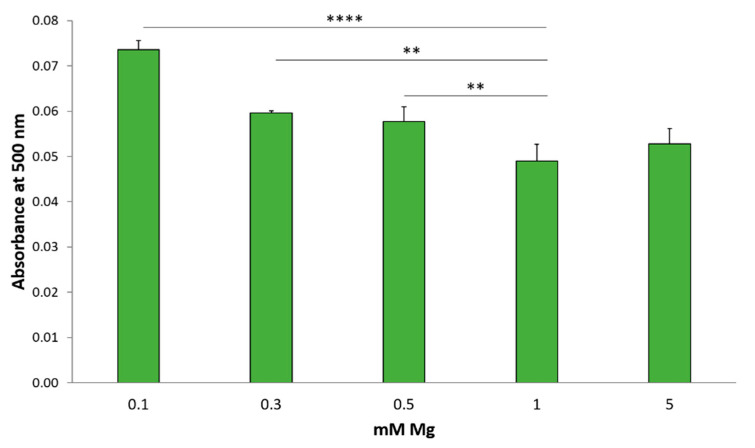
Low Mg induces lipid accumulation. HUVEC were cultured in different concentrations of Mg. 24 h later, the cells were stained with Oil Red O and solubilized. Absorbance was measured at 500 nm and normalized to the cell number. The experiment was performed three times in triplicates. ** *p* ≤ 0.01 and **** *p* ≤ 0.0001.

Since PPARγ, which is required for lipogenesis, and its transcriptional coactivator EDF-1 are upregulated in HUVEC by low extracellular Mg, we tested whether Mg-deficient cells deposit more lipids than the control. To this purpose, HUVEC were seeded in 24 wells, cultured in different concentrations of Mg for 24 h, fixed in paraformaldehyde (PFA) and stained using Oil Red O to detect neutral lipids [17]. As shown in Figure 5, higher amounts of lipids are detected in HUVEC exposed to media containing 0.1, 0.3 and 0.5 mM Mg than in the control. No differences are found between cells in 1 or 5 mM Mg.

## 3. Discussion

In cultured primary human EC, Mg deficiency upregulates EDF-1 through the induction of oxidative stress. Indeed, in accordance with previous studies [26,27], we found that culture in low Mg dose-dependently increases ROS production, an event that is reverted by exposure to the antioxidant NAC. The stimulation of oxidative stress by Mg deficiency is reported to be due to the reduced activity of antioxidant enzymes, among which are superoxide dismutase, glutathione peroxidase and catalase, as well as to the decrease in the levels of glutathione [30]. Accordingly, H_2_O_2_ mimics Mg deficiency in upregulating EDF-1. EDF-1 was proposed as a stress response protein by Jindra et al. [28], who showed that EDF-1 null Drosophila cells become hypersensitive to oxidative stress induced by H_2_O_2_ [28]. In this model, EDF-1 protects D-jun against oxidative modifications. Moreover, yeast EDF-1 mutants do not overcome nutritional stress [31] because EDF-1 is necessary for the activity of General Control Nondepressible (GCN) 4, a regulator of amino acid synthesis. Therefore, we hypothesize that in HUVEC, EDF-1 might be required to face oxidative stress, thus granting the activation of an adequate adaptive response to maintain cell viability. On these bases, we propose that the evolutionary conservation of EDF-1 might be explained by the evidence that it provides advantages under stress conditions. 

We also demonstrate that medium containing low Mg induces PPARγ, and this is not hampered by NAC, thus ruling out a role of ROS. Accordingly, H_2_O_2_ does not induce PPARγ. More studies are necessary to disclose the pathways involved in upregulating PPARγ in Mg-deficient HUVEC. PPARγ is a ligand-activated transcription factor that exerts a broad spectrum of biological functions and is fundamental in modeling inflammation and energy balance, including fatty acids handling and storage [32]. It is noteworthy that we found increased lipid content in HUVEC cultured in low Mg concentrations. This might result from alterations of endothelial metabolism induced by a low availability of Mg, which is implicated in hundreds of enzymatic reactions [33], and/or to increased transport of lipids from the extracellular environment. Moreover, it is likely that more lipids are synthesized in response to higher amounts of PPARγ and its transcriptional coactivator EDF-1. Interestingly, EC store fatty acids in lipid droplets as a protective measure against endoplasmic reticulum (ER) stress [34], which might be triggered by oxidative stress [35]. A concentration of 5 mM extracellular Mg does not modulate EDF-1 and PPARγ nor induce lipid deposition. It is noteworthy that Mg supplementation is beneficial in vascular disease [36]. *In vitro*, high concentrations of Mg stimulate endothelial proliferation and migration and protect endothelial cells against oxidative stress and inflammation [6,37]. 

In conclusion, we propose that Mg deficiency induces the deposition of lipids by upregulating PPARγ through an unraveled mechanism and EDF-1 through ROS. Due to the reduced dietary intake, subclinical Mg deficiency is common in industrialized countries and associated with increased cardiovascular risk [38]. Therefore, our studies offer novel insights into the complex mechanisms leading to endothelial dysfunction in Mg deficiency.

## 4. Materials and Methods

### 4.1. Cell Culture

Human umbilical vein endothelial cells (HUVEC) were obtained from the American Type Culture Collection (ATCC, Manassas, WV, USA) and cultured in medium M199 (Euroclone, Milano, Italy) containing 10% fetal bovine serum (FBS), 1 mM l-Glutamine, 1 mM Sodium Pyruvate, 1 mM Penicillin-Streptomycin, 5 U/mL Heparin and 150 µg/mL Endothelial Cell Growth Factor on 2% gelatin-coated dishes (Euroclone) [39]. To analyze the effects of different Mg concentrations, HUVEC were cultured in custom-made Mg-free medium (Thermo Fisher Scientific, Waltham, MA, USA) supplemented with Mg sulfate (MgSO_4_) to reach final concentrations ranging from 0.1 to 5 mM [40].

In some experiments, HUVEC were treated with hydrogen peroxide (H_2_O_2_) (200 μM) (Sigma Aldrich, St. Louis, MO, USA) for 30 min to induce oxidative stress, while N-acetylcysteine (NAC) (5 mM) (Sigma Aldrich) was used as an antioxidant.

### 4.2. Western Blot Analysis

HUVEC were lysed in 50 mM Tris–HCl (pH 7.4) containing 150 mM NaCl, 1% NP40, 0.25% sodium deoxycholate, protease inhibitors (10 µg/mL Leupeptin, 10 µg/mL Aprotinin, 1 mM PMSF) and phosphatase inhibitors (1 mM sodium fluoride, 1 mM sodium vanadate, 5 mM sodium phosphate). Protein concentration was assessed using the Bradford protein assay (Sigma Aldrich). Lysates (80 µg/lane) were separated on SDS–PAGE and transferred to nitrocellulose sheets at 400 mA for 2 h at 4 °C. The immunoblot analysis was performed using antibodies against EDF-1 (AVIVA Systems Biology Corporation, San Diego, CA, USA), PPARγ and actin (Santa Cruz Biotechnology, Dallas, TX, USA) [17]. Then, the nitrocellulose membrane was extensively washed and incubated with secondary antibodies labeled with horseradish peroxidase (Amersham Pharmacia Biotech Italia, Cologno Monzese, Italy). Immunoreactive proteins were detected with ClarityTM Western ECL substrate (Bio-Rad Laboratories, Hercules, CA, USA). Each experiment was performed 3 times and the Image J software (Version 1.52a, National Institutes of Health, Bethesda, MD, USA) was utilized to measure the ratio between the protein of interest and actin. We report the quantification of three different blots by ImageJ, and we show a representative blot.

### 4.3. ROS Production

For the detection of ROS, HUVEC were cultured in a 96-well black plate (Greiner bio-one, Frickenhausen, Germany). At the end of the experiments, cells were incubated for 30 min with 10 mM 2’-7’-dichlorofluorescein diacetate (DCFDA) solution (Thermo Fisher Scientific), while some samples were trypsinized and counted using a cell counter. The dye emission was monitored at 529 nm (λexc = 495 nm, λemm = 529 nm) using a Varioskan LUX Multimode Microplate Reader (Thermo Fisher Scientific) [41]. The amount of ROS production was normalized to the cell number. The results are the mean of three independent experiments performed in triplicate.

### 4.4. Oil Red O Staining

HUVEC were seeded in 24-well plates (Greiner bio-one, Frickenhausen, Germany) and cultured in different concentrations of Mg for 24 h. At the end of the experiment, cells were washed three times with PBS, fixed in PFA 10% for 30 min at room temperature, washed once again with PBS and then stained with 60% filtered Oil Red O stock solution (Sigma Aldrich) for 20 min. After extensive washing, Oil Red O was solubilized in 100% isopropanol and quantified by measuring the absorbance at 500 nm. The results are the mean of three independent experiments performed in triplicate.

### 4.5. Statistical Analysis

Data are reported as means ± SD. The data were normally distributed, and they were analyzed using one-way repeated-measures ANOVA. The *p*-values deriving from multiple pairwise comparisons were corrected by the Bonferroni method. Statistical significance was defined for a *p*-value ≤ 0.05. Regarding the figures, * *p* ≤ 0.05; ** *p* ≤ 0.01; *** *p* ≤ 0.001; **** *p* ≤ 0.0001.

## Data Availability

Data available in a publicly accessible repository. The data presented in this study are openly available in Dataverse at https://dataverse.unimi.it/dataverse/IJMS2021/.
